# Wide-Based Illumination and Detection in Functional Near-Infrared Spectroscopy for Enhanced Seizure Detection in Grey Matter

**DOI:** 10.3390/s25123627

**Published:** 2025-06-09

**Authors:** Netaniel Rein, Revital Shechter, Evgeny Tsizin, Mordekhay Medvedovsky, Michal Balberg

**Affiliations:** 1 Department of Neurology and Agnes Ginges Center for Human Neurogenetics, Hadassah Medical Organization, Jerusalem 9112001, Israel; netaniel.rein@mail.huji.ac.il (N.R.); evgenyt@hadassah.org.il (E.T.); mordehayme@hadassah.org.il (M.M.); 2 Faculty of Medicine, Hebrew University of Jerusalem, Jerusalem 9112001, Israel; 3 Neuro Electro Light Center (NELC)—The Joint Research Center of Hadassah Medical Organization and Holon Institute of Technology, Kalman Yaakov Man 2, Jerusalem P.O. Box 12000, Israel; revitalsh@hit.ac.il; 4 Faculty of Engineering, Holon Institute of Technology, Holon 5810201, Israel

**Keywords:** functional Near Infrared Spectroscopy (fNIRS), seizure detection, grey matter, EEG

## Abstract

Functional near-infrared spectroscopy (fNIRS) is a non-invasive method for monitoring brain activity by detecting hemodynamic changes. Studies have shown that it can identify ictal and pre-ictal hemodynamic variations, supporting its potential use as a complement to electroencephalography (EEG) in epilepsy monitoring. This study explores an expanded illumination and detection approach utilizing wide-based optodes and increased emitter–detector separation (EDS) to enhance fNIRS sensitivity to cortical hemodynamic changes while minimizing scalp contamination. A Monte Carlo simulation was designed to assess signal amplitude and sensitivity of fNIRS with varying emitter and detector diameters (1–15 mm) and EDS (30–50 mm). Signal strength, grey matter to scalp path ratio (GSPR), and percentage signal change per absorption coefficients (AC) variation were analyzed. Sensitivity to changes in AC of superficial and deep grey matter (SGM, DGM) and scalp was assessed. Increasing emitter and detector diameters substantially increased total detected photon packet weights, enabling practical use at larger EDS. Sensitivity to SGM AC changes tripled at 50 mm EDS, while GSPR increased by 80%, indicating reduced signal contamination from the scalp. Sensitivity to deep cortical hemodynamic changes also improved. Therefore, wide-based fNIRS optodes with increased EDS can enhance seizure-related hemodynamic detection, potentially improving epilepsy diagnostics.

## 1. Introduction

### 1.1. Epilepsy and Electroencephalography (EEG)

Epilepsy is one of the most prevalent chronic neurological disorders and is characterized by an enduring predisposition to recurrent, unprovoked seizures, with significant neurobiological, cognitive, psychological, and social consequences [1]. The most common tool for detecting seizure activity is scalp electroencephalography (EEG). Despite its utility, affordability, safety, and simplicity, EEG has limitations, including the need for synchronized neuronal activity for detection [2,3,4], low signal-to-noise ratio [5], and rapid spread of ictal activity, limiting the precise localization of the seizure onset zone (SOZ) [5]. EEG is further limited by restricted depth sensitivity, which is caused by several factors including attenuation of signals by the skull and scalp [6], low signal-to-noise ratio (SNR) of deep sources [7], and the orientation of electrical dipoles in the sulcal regions [8,9].

### 1.2. Functional Near-Infrared Spectroscopy (fNIRS) and Epilepsy

Functional near-infrared spectroscopy (fNIRS) is a non-invasive technique measuring brain activity via changes in oxyhemoglobin (HbO) and deoxyhemoglobin (HbR) levels, offering portability and long-term monitoring in natural settings. The technique is based on emitting light at different near-infrared wavelengths and measuring the intensity of light that reaches a detector placed at a distance (typically 30 mm). Changes in this intensity mainly correspond to changes in the absorption of light by hemoglobin and its derivatives. Each derivative, mainly HbO and HbR, has a different wavelength-dependent absorption coefficient. The total absorption at each wavelength depends on the volume and concentration of absorbers (i.e., the hemoglobin molecules) in the illuminated tissue and the ratio between HbO and HbR. Accounting for the difference between HbR and HbO can be performed using more than one wavelength in the measurement. For the purpose of the simulation we chose one wavelength (630 nm). Since the volume of blood within the tissue’s microvasculature changes to address variations in neuronal activity via neurovascular coupling, it can be assumed that changes in the detected energy correspond to changes in activity [10,11,12]. Due to the absorption of light by hemoglobin (Hb), one of the main sources of noise of fNIRS is hemodynamic fluctuations in the scalp [13]. In epilepsy, fNIRS complements EEG by detecting hemodynamic changes, associated with the significant neuronal activity during seizures, potentially aiding in seizure focus localization. Studies have shown its utility in identifying hemoglobin variations linked to seizures [14,15,16] and interictal discharges [17].

Similarly to EEG, detection of peri-ictal changes using fNIRS is limited to the outer 10–15 mm of the intracranial space [18] and requires an array of channels with emitter–detector separation (EDS) of 30–33.5 mm [19]. Attempts to overcome the depth limitation of fNIRS include advanced computational methods [20] and recently an intracranial fNIRS system composed of an implanted optode and an optical anchor bolt was proposed [21]. These methods require either complex calculations or invasive devices.

### 1.3. Expanded Illumination and Detection with fNIRS

In this paper, we propose a novel wide-based illumination and detection fNIRS system designed to improve scalp-based seizure detection by enabling more light to reach the grey matter vasculature while simultaneously reducing contamination by the scalp’s hemodynamic fluctuations using longer EDSs. Unlike EEG, fNIRS is not subject to limitations relating to the direction of the electrical dipole. It does not passively measure a signal emitted by the cortex but rather emits a signal and measures its absorption in the tissue. This enables control of the strength of the signal, and although it is limited by safety constraints, there is a wide safety range between the energy used and that which poses a risk of tissue injury [22]. To allow for an increase in the emitted energy and overcome the limitations dictated by safety considerations, we propose to increase the size of the standard point optodes (~1 mm) to wide-based illumination and detection optodes with a diameter of 5–15 mm or even more.

Increasing the diameter of the emitter while keeping the energy per area similar would yield a stronger total signal without the increased risk of tissue injury as safety limitations are defined in power or intensity per unit area [23]. This design could be combined with novel materials [24] that could increase energy efficiency and further reduce the risk of thermal injury. The increase in signal would enable an increase in the maximum EDS with a usable signal, which could yield an increase in the ratio between the signal from the GM and the hemodynamic noise from the scalp. It may additionally increase the depth of detection of peri-ictal hemodynamic changes, creating a simple, portable, non-invasive fNIRS tool that could detect seizures in areas previously inaccessible when scalp-positioned portable systems are used. These areas include deep sulci such as the interhemispheric fissure and, in the absence of hair (or after shaving), the Rolandic and Sylvian fissures and the inferior surface of the cortex. The main limitation of detection in this case would be the ratio between the signal from deep tissues and that from the more superficial layers.

**Figure 1:**
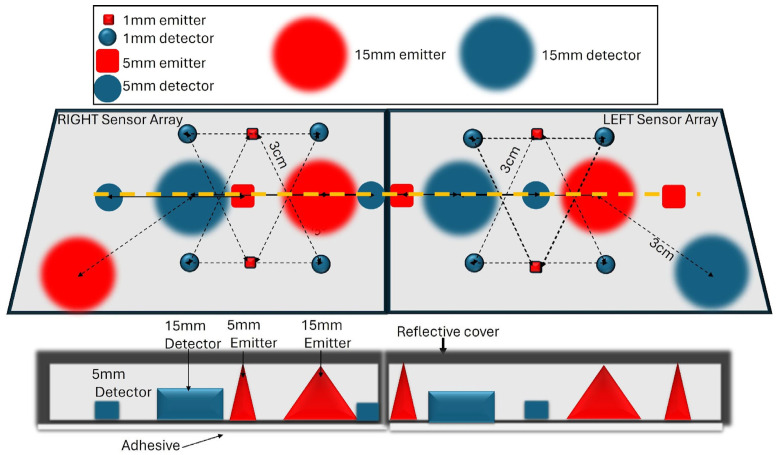
A proposed configuration of the proposed sensor. (**Top**) A two-dimensional top view of an array of emitters and detectors embedded in a scattering layer that enables expanded illumination, integrated with a reflective layer preventing loss of signal to the environment. Emitters and detectors of various diameters are arranged to detect light traveling through different layers. (**Bottom**) A cross-section of the sensor, along the orange dotted line, illustrating the expanding cone of light (in red) generated by a source emitting into the scattering layer, resulting in wide-based illumination, and the different detector sizes.

The model we propose for wide illumination consists of a 5–10 mm thick layer of scattering material, such as white thermoplastic polyurethane (TPU), with thickness adjustable to achieve the desired level of scattering. The outer surface is coated with a reflective polymer-encapsulated aluminum layer, which will house the embedded emitters and detectors. The light emitted from certain emitters would traverse the scattering layer en route to the scalp which will expand the beam of light, creating a de facto wide-based emitter (Figure 1). This method provides a simple and affordable way to create wide-based emitters up to several centimeters in diameter. It can also be adapted modularly, enabling a single large emitter to function as alternating smaller emitters, thus creating more channels and information.

Another potential method for enhancing signal strength is to increase the detection area by applying large area silicon photodiodes up to 15 mm in diameter. This method could be used in tandem with increased illumination, and it has the advantage of increasing the magnitude of the signal detected without the increased risk of tissue injury.

To assess the utility of expanded illumination and detection, a Monte Carlo simulation was created, using the different areas of emitters and detectors. The simulation was designed to compare the detection of signal changes and the specificity for the GM signal of wide-based illumination and detection and their combination with traditional scalp fNIRS.

Another potential method for enhancing signal strength is to increase the detection area. This method could be used in tandem with increased illumination areas, and it has the advantage of increasing the magnitude of the signal detected without the increased risk of tissue injury.

To assess the utility of expanded illumination and detection, a Monte Carlo simulation was created. The simulation was designed to evaluate changes in signal and SNR with wide-based illumination and detection and their combination compared to traditional scalp fNIRS.

## 2. Methods

### 2.1. Structure of Monte Carlo Simulation

**Figure 2:**
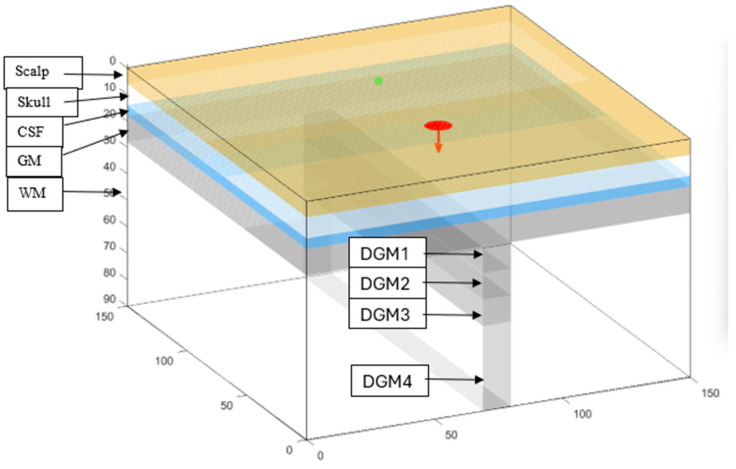
A 3D model of the Monte Carlo simulation volume with a disk-shaped emitter (red) and a detector (green) placed on the scalp at 50 mm emitter–detector separation. The head is simulated with 5 layers: scalp, skull, cerebrospinal fluid (CSF), grey matter (GM), and white matter (WM). Segmented deep GM perpendicular to superficial grey matter simulating sulcal deep grey matter divided into 4 layers. The layers are deep grey matter 1 (DGM1), DGM2, DGM3, and DGM4 and are 28–38 mm, 38–48 mm, 48–58 mm, and 58–90 mm deep from the head surface, respectively.

A Monte Carlo simulation (see Supplementary Materials for code) was constructed to examine the benefits of expanded illumination and detection surfaces in fNIRS. The simulation was of a 90 × 150 × 150 mm^3^ slab model of the head with nine different layers (Figure 2) (Monte Carlo eXtreme (MCX) (https://mcx.space), MATLAB^®^ 2022b). The first five layers were horizontal and represented the scalp, bone, CSF, GM, and white matter (WM) and were 6, 8, 4, 10, and 62 mm thick, respectively. Additionally, 4 layers were placed perpendicular to the superficial layer of GM and into the WM and represented sulcal deep GM. The sulcal GM was divided into four layers: deep GM1 (DGM1), DGM2, DGM3, and DGM4. The first three layers (10 × 10 × 100 mm^3^ each) extended sequentially deeper into the WM at depths of 28–38 mm, 38–48 mm, and 48–58 mm. The fourth layer (10 × 32 × 100 mm^3^) extended 58–90 mm deep. This segmentation allowed for the simulation of different optical properties at varying depths.

The slab model was selected for its simplicity and has been successfully utilized in previous fNIRS MC simulations [21,25]. While a prior simulation study [26] identified potential inaccuracies when using a slab model instead of a more anatomically detailed brain model, these discrepancies were primarily observed at small emitter–detector separations (EDSs) of 8.4 mm [26]. The differences were considerably reduced at more standard separations of 30–35 mm, supporting the slab model’s suitability for our application.

**Figure 3:**
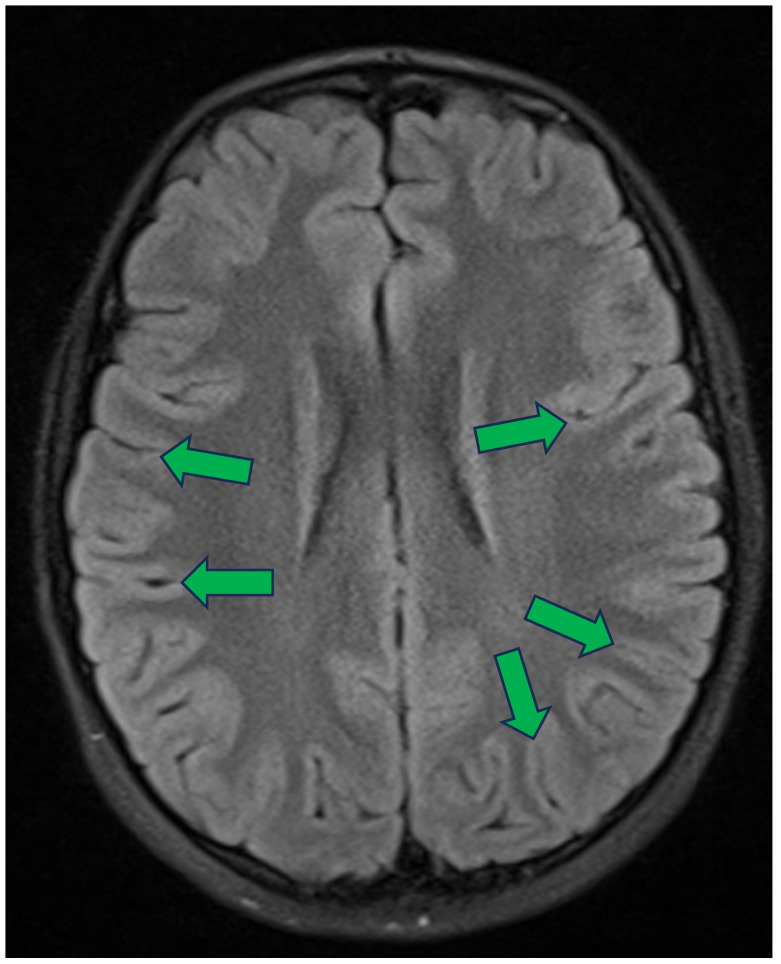
Axial section of fluid-attenuated inversion recovery (FLAIR) sequence of brain MRI of a young girl [28]. Numerous sulci are visible, with most appearing to form an angle close to a right angle relative to the skull and the scalp. Green arrows ending at the deepest visible point of several sulci oriented perpendicularly to the surface of the head.

The perpendicular DGM structure in the middle was added to the simple slab model to simulate the penetration of grey matter layers in sulci and fissures. Its geometry was based on the minimal width of sulcal cerebrospinal fluid (CSF) spaces observed in young patients [27] as well as the common observation that most sulci are oriented perpendicularly to the scalp and skull (Figure 3) [28]. This choice reflects the typical age group undergoing drug-resistant epilepsy evaluations, for whom these anatomical characteristics are most relevant.

The optical properties of the layers (Table 1) were based on an article by Fang et al. (2010) [29], for a wavelength of 630 nm, with the reduced scattering coefficient of the white matter (WM) selected based on our recent work [21] that examined the effect of WM scattering on fNIRS signals.

### 2.2. Emitter and Detector Parameters

First, we note that in the simulation we measured the detected energy of the light and not intensity as measured in real-life settings. To account for this, we assume that all the detectors in the simulations are identical, except for their area. As shown in Figure 2, an emitter and a detector were placed above the scalp layer, along the longer dimension of the sulcal GM. Both were directed intracranially in all simulations, and light was emitted in a direction perpendicular to the plane of the scalp layer. Emitters were designed to emit 5 × 10^7^ × (diameter of the emitter in mm)^2^ packets of photons to simulate a disk emitting a constant amount of photon packets per area in all simulations. Initially, emitters of increasing diameter (range 1–5 mm) were simulated, and the detector was set to a 1 mm diameter. This was followed by simulating a detector of increasing size (diameter range 1–5 mm) with the emitter’s diameter set to 1 mm and comparing the results of increasing diameter of emitters and detectors.

Simulations with increasing diameter of detectors (1–15 mm) for EDSs of 30, 40, and 50 mm were run to assess the impact of wide-based optodes on the detected photon packet weights and sensitivity to changes in the optical properties of different layers. Finally, both the emitter and the detector were simulated with a 5 mm diameter to assess the utility of the combined enlargement of both components of the system.

Simulations were initially run with an EDS of 30 mm, which is considered optimal for standard fNIRS systems [18], while systematically increasing optode diameters. Subsequently, the EDS was extended to 40 and 50 mm as was performed in previous studies [18] to evaluate the impact of greater separation on simulation metrics, given the expected signal enhancement with larger optodes.

In several configurations, the sensitivity to changes in the absorption coefficient (AC) was assessed by increasing the AC in the SGM, DGM1, and the scalp by up to 25%. These changes simulate changes in the light absorption by hemoglobin that correspond to substantial changes in blood volume (as a surrogate to blood flow) in each layer.

### 2.3. Metrics of the Simulation

The metric of energy was defined as the weight assigned to a single photon packet upon launch. To quantify the detected energy, we reran each simulation using only the detected photons and calculated the difference between the total energy launched and the total absorbed energy across all voxels. Since all exiting photons in the repeated simulation reached the detector, this difference represented the total detected energy.

This approach was possible because MCX operates as a pseudorandom number generator (PRNG) [30] and stores the seeds of detected photons, allowing for precise re-simulation.

For each simulation the following parameters were recorded: (1) total detected photon packet weights; (2) number of detected photons packets; (3) the number of photons packets traversing each layer; (4) the average partial path length per layer (in mm).

As a realistic system with reasonable optical power cannot be simulated using an MC model within a reasonable computation time, we decided to compare the simulated output to that obtained when the simulation’s parameters were set to simulate conventional (standard) fNIRS optodes with a 1 mm diameter detector placed 30 mm from a 1 mm diameter emitter. Since standard fNIRS configurations have been shown to detect pre-ictal and ictal hemodynamic changes in both previous studies [31] and our own data (Appendix A), their simulation output was used as a benchmark for evaluating all other configurations.

The standard measured signal (SMS) was defined as the energy detected by a standard fNIRS system, with an AC (μ_a_) set to those of Table 1, simulating the typical signal measured in conventional fNIRS systems. In order to measure the SMS accurately and since MCX employs PRNG, its assessment required running simulations with different seeds, as identical simulations would produce the same results [30]. To assess the repeatability and standard deviation, the simulation was run 50 times with different seeds with the optodes positioned at slightly (1 voxel apart) different locations along the scalp and parallel to the longer direction of the DGM while all other parameters remained unchanged. Emitters and detectors were positioned sufficiently far from the simulation boundaries (minimum distance of 35 mm) to prevent confounding effects from photon absorption at the edges. SMS was defined as the mean energy across these simulations.

To simulate the effect of a seizure on the detected signal, the change in the detected energy resulting from an increase in the AC (μ_a_) by 25% (from 0.020 to 0.025 mm^−1^) was calculated. The baseline signal for each system configuration was calculated by running a simulation using the default AC specified in Table 1. Changes in the detected signal were expressed as relative deviations from this baseline (in %), calculated per incremental increase in the AC of a specific layer, while the AC of other layers and all other optical properties were unchanged. The simulated change in the AC of the GM was based on reported ranges of cerebral blood volume (CBV) variation during seizures. Previous studies have shown that seizures can induce CBV increases of 16–55% in PET imaging [32] and approximately 21% in fMRI [33]. Since hemoglobin is the primary chromophore in brain tissue affecting AC in fNIRS systems [34], the simulation modeled up to a 25% increase in GM AC to reflect peri-ictal CBV changes within this physiologically relevant range.

To ensure that the signal change was due to absorption rather than random noise, the percentage change in signal was calculated at 50 different positions using a method similar to the one described above. The mean signal change and standard deviation were then computed.

The comparison of different simulations with variable ACs was based on the following: since MCX utilizes a PRNG, repeated simulations with similar seeds yield identical results, as stated above. Additionally, because MCX launches photon packets rather than individual photons, when equal numbers of packets are detected in two simulations with similar seeds but different absorption coefficients, the difference in the total detected energy corresponds to the amount of energy absorbed due to the change in the AC.

To evaluate the impact of different emitter and detector positions and sizes on their ability to detect significant hemodynamic changes, the following metrics were used: (1) the total energy detected was compared to SMS to determine whether the signal was strong enough for practical use (i.e., detectable). (2) Sensitivity to changes in absorption was defined as the change in detected energy per change in the AC of the relevant layer. (3) Grey matter to scalp path ratio (GSPR) was calculated as the ratio between the total photon path length through SGM and total photon path length through the scalp. The total partial path length through a specific layer was calculated by multiplying the number of photon packets traversing the layer by their average partial path length (APPL) within it (Equation (1)). Both the APPL and the number of photon packets passing through each layer are outputs generated by the MCX model. This calculation was performed separately for the SGM and the scalp. The ratio between these values yielded the GSPR (Equation (2)).

GSPR, introduced in an earlier study [21], serves as a metric for comparing the relative sensitivity of fNIRS signals to grey matter versus scalp contributions, the latter being a primary source of hemodynamic noise in brain activity measurements [35,36]. The rationale behind this metric is as follows: fNIRS measures changes in Hb concentration based on the amount of light absorbed as it travels through tissue. The longer the optical path through a given tissue, the greater the likelihood that light will be absorbed by Hb within that tissue. By comparing the total photon path length in one tissue to that in another, we obtain a metric that reflects the relative sensitivity to changes in Hb concentration across tissues.

(1) Total layer path length = (Layer APPL) × (photons packets traversing layer)

(2) GSPR = (Total grey matter path length)/(total scalp path length) 

The GSPR of the simulated standard fNIRS system (30 mm emitter–detector separation and 1 mm wide optodes) was calculated at 50 different positions, following a procedure similar to that used for the SMS calculation. The mean and standard deviation of the GSPR were then computed to ensure that variations observed with different optode sizes and positions exceeded the expected range of random noise.

## 3. Results

The mean SMS for 50 simulations with different seeds was 5.18 photon packet weights with a standard deviation of 0.36 photon packet weights.

### 3.1. Simulation with Increased Diameter of Emitter and Detector

**Figure 4:**
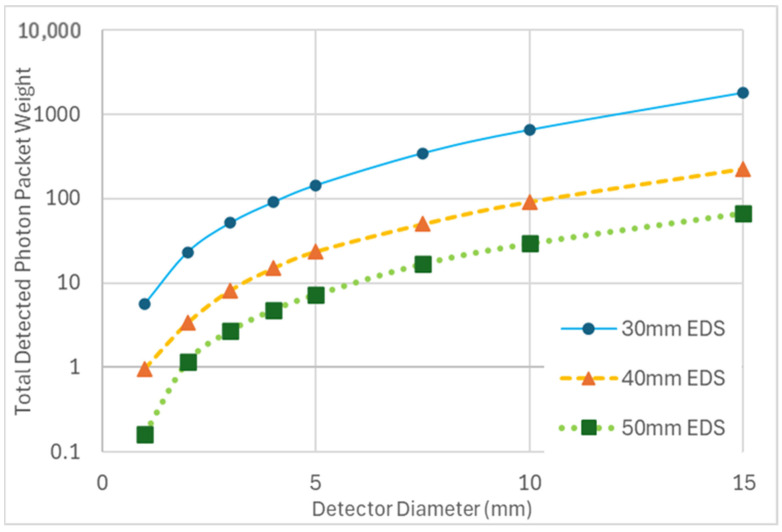
The plot illustrates changes in total detected photon packet weight as a function of detector diameter, with increasing emitter–detector separation (EDS). In all simulations, the emitter diameter is fixed at 1 mm.

Increasing the diameter of either the emitter or the detector yielded comparable and dramatic increases in the total detected energy (Table 2). This increase in total detected energy did not result in an increased sensitivity to change in the AC of the SGM. A comparable decrease of ~3% in total energy per a 25% increase in SGM AC was observed for diameters of 1 to 5 mm of either emitter or detector at a 30 mm EDS (Table 2). Increasing the diameter of the detector at EDSs of 40 and 50 mm also yielded an increase in the total detected energy (Figure 4). However, the increased diameter at various EDSs affected only minor changes in percent decrease in total energy per increase of 25% in SGM AC, with no clear trend. This indicates that the diameter of either optode correlates with total energy but not with sensitivity to changes in SGM AC.

The decrease in energy is measured per 25% increase in the absorption coefficient of superficial grey matter. The unchanged optode (emitter for the simulations of increasing detector diameter and detector for the simulations of increasing emitter diameter) has a diameter of 1 mm, with an emitter–detector separation of 30 mm.

### 3.2. Impact of Increased Emitter–Detector Separation on Sensitivity to Changes in SGM AC

Repeating the simulation 50 times with different seeds to measure the percent change in signal per 25% increase in SGM AC for a standard system (30 mm EDS, 1 mm diameter optodes) resulted in a mean signal decrease of 2.78% with a standard deviation of 0.22%. This consistency indicates that the observed signal change is not attributable to random noise.

**Figure 5:**
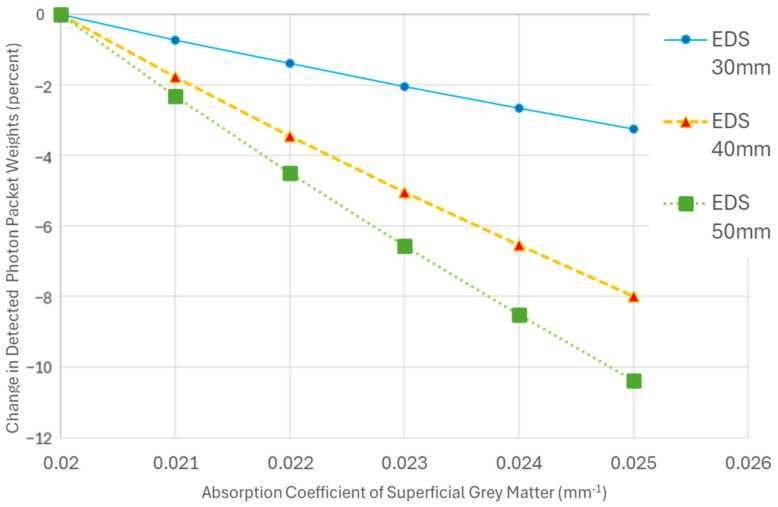
A plot illustrating the percent change in the detected photon packet weights from baseline as the absorption coefficient (AC) of the superficial grey matter (SGM) increases. Each trend line represents simulations with a fixed emitter–detector separation (EDS) and diameter while varying the AC of the SGM. The baseline for each trend line is set to the detected photon packet weights when the AC of SGM is 0.02 mm^−1^.

The sensitivity to changes in the AC of the SGM increased with EDS (Figure 5). Specifically, for a 25% increase in SGM AC, at a 50 mm separation, the change in the detected energy was more than 3 times higher than at a 30 mm separation. With 5 mm diameter emitters and detectors, the absolute change in signal increased from 3.2% at a 30 mm EDS to 10.3% at a 50 mm EDS for a 25% change in AC of the SGM. This finding confirms that larger emitter–detector distances enhance the ability of the system to detect significant hemodynamic changes in the SGM.

It should be noted that the detected photon packet weights at a 50 mm EDS with 1 mm optodes dropped to 0.16 photon packet weights, amounting to only ~3% of the SMS, rendering the signal practically unusable. In contrast, with 5 mm optodes, 176 photon packet weights were detected—more than 30 times greater than the SMS (Table 3).

**Figure 6:**
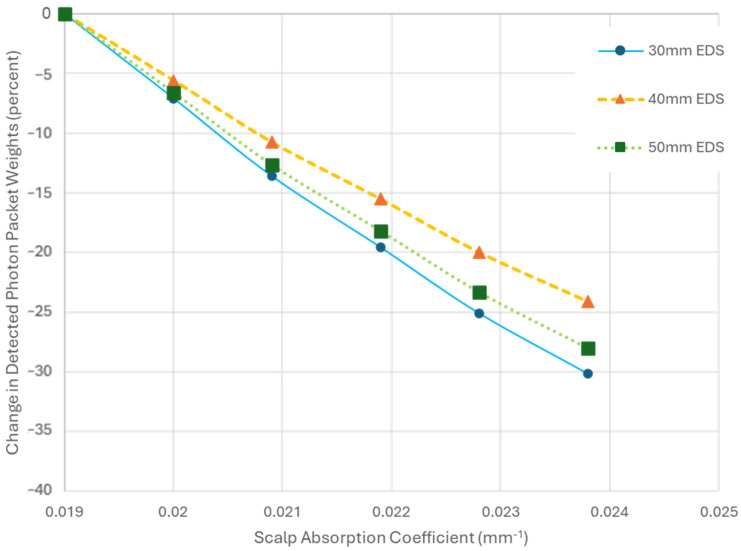
A plot showing the change in the detected photon packet weights from baseline as the absorption coefficient (AC) of the scalp increases. Each trend line represents simulations with fixed emitter–detector separation (EDS) and diameters while varying the scalp AC. The baseline for each trend line corresponds to the detected photon packet weights when the scalp AC is set to 0.019 mm^−1^.

Unlike the sensitivity observed for changes in the AC of SGM for different EDSs, the effect of variations in scalp AC at 30 mm on the detected energy does not vary much with increasing EDS (Figure 6).

### 3.3. Impact of Emitter–Detector Separation on Superficial Grey Matter to Scalp Path Ratio (GSPR)

The GSPR was calculated across 50 simulations using parameters representative of a standard fNIRS system (1 mm wide optodes and a 30 mm emitter–detector separation), yielding a mean GSPR of 0.848 with a standard deviation of 0.053.

**Figure 7:**
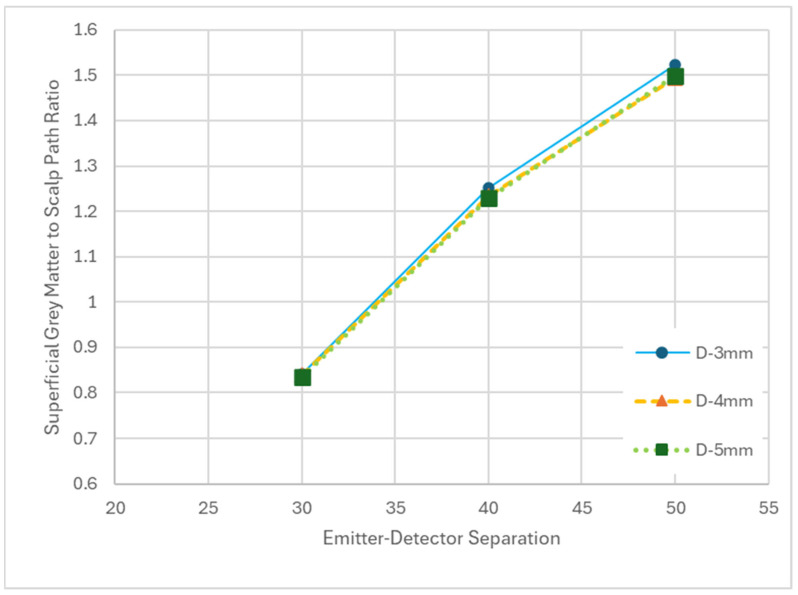
Plot of superficial grey matter to scalp path ratio (GSPR) as it relates to the emitter–detector separation (EDS) for variable detector diameters (D).

Increasing EDS yielded an increase in GSPR while increasing emitter and detector diameter yielded minimal variations in GSPR with no clear trend (Figure 7). The increase in GSPR from 30 mm EDS to 50 mm was ~80%. Although optode diameter did not have an important impact on GSPR, it did impact the total photon packet weights detected (Table 2), thus enabling the useability of the system at a greater EDS as noted above.

### 3.4. Impact of EDS on Sensitivity to Changes in the AC of DGM1

Changes in the AC of DGM1 of up to 25% in all conditions including EDSs of 30, 40, and 50 mm and emitter and detector diameters of 1, 5, and even 15 mm caused a change no greater than 0.1% of the total detected photon packet weights.

Similarly to the SGM, an increase in EDS from 30 mm to 50 mm with optodes set to 5 mm diameter caused an increase of more than 3-fold in the change of the total detected photon packet weights for an increase of 25% in the AC of DGM1.

## 4. Discussion

Using an MC simulation, we demonstrated the effectiveness of wide-based illumination and detection in enhancing the detected NIR signal magnitude. The system’s sensitivity to hemodynamic changes—measured by the relative change in the detected photon packet weights due to variations in the AC of the grey matter—was influenced by increased EDS but remained unaffected by changes in optode diameter. In addition, the ratio between the superficial grey matter to scalp paths (GSPR) increases with EDS, indicating a reduced sensitivity to scalp hemodynamics, but is not affected by the optode’s area. However, increasing the optode’s diameter was crucial for maintaining a detectable signal at greater EDSs.

The increase of more than three orders of magnitude in the detected energy gained by increasing the diameter of the emitter and the detector from 1 mm to 5 mm at 50 mm separation and creating a signal over 30 times stronger than the SMS for a 50 mm EDS renders the detection of a signal possibly even more reliable than standard fNIRS channels with optodes 30 mm apart.

Increasing the diameter of either the emitter or detector produced comparable results in terms of total detected photon packet weights and sensitivity to changes in the AC of the SGM. Since enlarging the detector poses no safety concerns, it is logical to prioritize this approach over increasing the emitter size. However, given the limited surface area of the head and the fact that safety concerns are mitigated by maintaining constant energy output per unit area, increasing both the emitter and detector by a lesser amount may be the most practical solution without introducing significant safety risks. Another theoretical approach to increasing the signal is to expand the number of emitters and detectors, thereby enhancing signal detection. While technically feasible, this solution would introduce greater mechanical complexity, require a more intricate arrangement, and likely incur higher costs, making it a less practical option.

Additionally, as the frequency of peri-ictal hemodynamic fluctuations detected in recent work [37] is in the order of 0.1–0.01 Hz (Appendix A), detection of hemodynamic activity at these frequencies permits the use of a relatively low sampling rate (in the range of ~0.2 Hz according to the Nyquist theorem). This low-frequency sampling provides an additional strategy for reducing the potential risk of injury to the tissue.

Wide-based illumination and detection have another advantage as their wide contacts make the signal less susceptible to artifacts from focal hemodynamic fluctuations in the scalp near either optode. Noise from such variations would be averaged from the entire optode area, thereby limiting its effect on the signal.

A known limitation of wide-based optodes with extended EDS arises from the larger volume sampled, compared to that sampled by smaller elements at the same separation. This, and increasing EDS, could reduce the measurement sensitivity since, as was true for noise, the detected signal would average changes in hemodynamic sources from the entire volume sampled. This limitation can be practically addressed by leveraging the modularity of our system (Figure 1) and alternating between wide-based optode channels and standard fNIRS channels. By doing so, we can harness the advantages of both configurations while mitigating their respective limitations. Furthermore, this limitation is less of a concern when detecting seizure activity compared to cognitive and motor activity. Conventional fNIRS systems are designed for detecting hemodynamic response functions (HRFs) that are mostly associated with cognitive [38,39,40] or motor activation [41,42]. Seizures activate large areas of the cortex [3], causing slow, large-amplitude hemodynamic fluctuations (see Appendix A). These significant hemodynamic changes are reflected by blood flow alterations in intracranial arteries supplying large cortical areas, detectable by transcranial Doppler [43,44].

Several previous studies have reported an increase in total hemoglobin during seizures [14,16,45]. A positron emission tomography (PET) study demonstrated a focal increase in cerebral blood volume (CBV) following electrical discharges, ranging from 16% to 55% [32]. Since hemoglobin (Hb) is the primary chromophore in brain tissue, it is reasonable to assume that such an increase in blood flow would lead to a proportional increase in the AC of the relevant tissue. While the exact magnitude of CBV change remains uncertain, the primary goal of this study was to compare different system parameters, making the exact simulation of AC changes unnecessary. This reasoning applies to the detection of both HbO and HbR at various NIR wavelengths, as simulations of progressively increasing AC consistently showed a gradual decrease in total signal across all configurations and were referenced to the SMS which was defined using the same optical coefficients. Additionally, a previous study [46] demonstrated that the spectral dependance of the of the absorption and scattering coefficients was reduced with wide-based optodes in fNIRS.

The increase in both the percentage change in signal per change in SGM AC and the GSPR, without a significant change in the percentage of signal per change in scalp AC with increasing optode separation, demonstrates the system’s improved sensitivity and specificity. The 200% increase in signal change relative to SGM AC, combined with the higher total signal from larger optodes, potentially enhances the sensitivity to ictal hemodynamic changes. GSPR, which reflects the ratio of photon packet paths through SGM versus the scalp, indicates the signal’s relative susceptibility to the target tissue (SGM) and the primary noise source in scalp-based fNIRS (scalp). The 80% increase in GSPR at a 50 mm optode separation compared to the standard 30 mm, along with the unchanged relative change in the total signal per change in the scalp’s AC, highlights the system’s improved resistance to hemodynamic noise from the scalp.

Detection of ictal hemodynamic changes in the brain has been shown using fMRI [47] and SPECT [48,49], but these methods have several shortcomings. The detection of ictal activity during fMRI or EEG-fMRI scans is usually fortuitous and scarce, as seizure onset is usually unpredictable and movement artifacts during seizures significantly impair data quality [47]. The short duration of monitoring limits the utility of fMRI in the detection of seizures as extended monitoring is usually required to detect and localize ictal activity [50]. For these reasons fMRI is normally used for detection of BOLD changes that correlate with interictal activity, and data of hemodynamic changes during seizures is relatively limited. In addition, both SPECT and fMRI are relatively cumbersome and costly and are susceptible to motion artifacts. Current fNIRS systems provide continuous, affordable, and ecological monitoring for extended periods and are coupled with EEG to attain hours and even days of combined electrical and hemodynamic monitoring. Additionally, fNIRS has been shown to detect pre-ictal changes preceding electrographic ictal findings by more than a minute [31] (Appendix A). Considering all these advantages, we believe that fNIRS systems should be integrated into the workup of patients with epilepsy and wide-based optodes with increased EDS could be used to increase sensitivity to ictal hemodynamic changes.

The detection of hemodynamic changes in sulcal grey matter was assessed by measuring signal variations in response to changes in the AC of DGM1. Although increasing the optode separation from 30 mm to 50 mm resulted in a ~200% relative increase in detected changes, the maximum signal change across all configurations remained ~0.1%. This finding aligns with previously reported [18] depth limitations of scalp-based fNIRS, reinforcing the need for creative solutions—such as the invasive monitoring or advanced algorithms mentioned above—to improve fNIRS-based detection of sulcal grey matter hemodynamic changes.

Several factors are known to influence fNIRS signal quality. Anatomical variability, such as regional and interindividual differences in scalp and skull thickness, can alter signal strength, with the scalp having a slightly greater impact on attenuation [51]. Hair characteristics also play a role as lighter hair tends to allow better signal quality, likely due to reduced absorption and scattering of near-infrared light [52]. Although fNIRS is generally more robust to motion artifacts compared to fMRI or PET [40,53], such artifacts—particularly those caused by optode displacement—can still introduce significant noise [54,55]. These issues must be addressed in systems using wide-based optodes; however, the broader surface area may offer greater stability against localized motion artifacts and reduced sensitivity to slight changes in optode angle or interference from hair. This study has the limitations inherent to all simulations in that it is based on certain model assumptions and that possible unknown confounding factors (e.g., sources of noise) were not accounted for by the simulations. These issues will be addressed when the sensor patch is fabricated and in vivo experiments are conducted to further examine these findings. Coupling optodes to the skin is challenging for large-area elements. In particular, the design of wide-based emitters and detectors is not ideal for coupling light to the scalp via hair cells and hair follicles. As such, the described configuration is more suitable for hairless regions of the head, such as the forehead or around the ears. When monitoring bald subjects, multiple locations can be accommodated. However, coupling flat elements to curved surfaces remains a challenge that limits the area of the optodes. Flexible elements, such as organic photodetectors [47,56] or flexible support structures, may be used to address these constraints.

## 5. Conclusions

In this study, using an MC simulation of a head model, wide-based illumination and detection with scalp-positioned fNIRS demonstrated a detectable optical signal at greater distances between emitter and detector, thus enabling a substantially increased sensitivity and specificity of the system to cortical hemodynamic changes associated with epileptic seizures. These findings could enhance the utility of fNIRS in epilepsy monitoring and warrant corroboration in future in vivo studies.

## Figures and Tables

**Table 1:** Optical properties of the layers of the head model in the Monte Carlo simulation.

	Thick-Ness (mm)	Absorption Coefficient μ_a_ (mm^−1^)	Scattering Coefficient μ_s_ (mm^−1^)	Reduced Scattering Coefficient μ_s′_ (mm^−1^)	Anisotropy (g)	Refraction Index (n)
Scalp	6	0.019	7.8	0.86	0.89	1.37
Skull	8	0.019	7.8	0.86	0.89	1.37
CSF	4	0.004	0.009	0.001	0.89	1.37
Grey matter	10	0.02	9.0	0.99	0.89	1.37
White matter	72	0.08	8.5	0.85	0.9	1.37

**Table 2:** Comparison of the effects of changes in detector and emitter diameter from 1 mm to 5 mm on total detected photon packet weights.

Optode Diameter (mm)	Photon Packet Weights per Detector Diameter (mm)	Photon Packet Weights per Emitter Diameter (mm)	Percent Change in Detected Photon Packet Weights per Detector Diameter (%)	Percent Change in Detected Photon Packet Weights per Emitter Diameter (%)
1	5.56	5.56	2.74	2.74
2	22.9	23.2	3.01	3.11
3	51.4	50.0	3.22	3.34
4	90.1	88.9	3.29	3.35
5	144	142	3.31	3.22

**Table 3:** Total detected photon packet weights with increasing emitter–detector separation (EDS).

EDS	Detected Photon Packet Weights for Diameter of 1 mm for Both Emitter and Detector	Detected Photon Packet Weights for Diameter of 5 mm for Both Emitter and Detector
30 mm	5.18	3733.4
40 mm	0.958	558.7
50 mm	0.161	176.0

**Figure A1:**
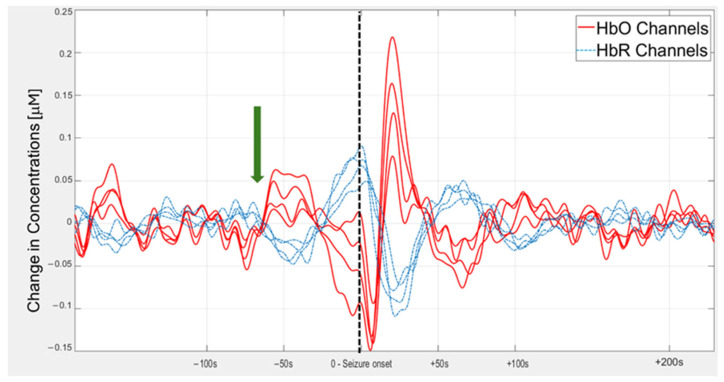
Preliminary results from a clinical study of combined fNIRS sensing and video-electroencephalography (VEEG) in patients with drug-resistant epilepsy. The study was approved by the institutional review board (study number 0068-22-HMO). In four channels (30 mm separation between emitter and detector), located over the left fronto-temporal area, the change in the concentrations of oxyhemoglobin (HbO) (solid red lines) and deoxyhemoglobin (HbR) (dashed blue lines) is presented as a function of time, relative to the electrographic seizure onset. The electrographic seizure onset is identified by an expert based on the VEEG signals and is marked by a dashed black line (time = 0). The data from the fNIRS channels, positioned around the seizure onset zone over the left fronto-temporal area, show a marked increase in amplitude of slow frequency oscillations (around 0.01 Hz) that begin approximately a minute before seizure onset (green arrow).

## Data Availability

The raw data supporting the conclusions of this article will be made available by the authors on request.

## Supplementary Materials

The code for the simulation is provided in the following link: https://github.com/Netaniel2/MC-simulation-fNIRS/blob/main/Disk_emitter_code.m (accessed on 14 May 2025).

## Abbreviations

The following abbreviations are used in this manuscript:

fNIRS	Functional Near-Infrared Spectroscopy
EEG	Electroencephalography
SMS	Standard Measured Signal
SNR	Signal to Noise Ratio
rSNR	Reference SNR
fMRI	Functional Magnetic Resonance Imaging
BOLD	Blood Oxygen Level Dependent
GM	Grey Matter
DGM	Deep Grey Matter
WM	White Matter
CSF	Cerebrospinal Fluid
MC	Monte Carlo
